# Transcriptome analysis of female western flower thrips, *Frankliniella occidentalis*, exhibiting neo-panoistic ovarian development

**DOI:** 10.1371/journal.pone.0272399

**Published:** 2022-08-01

**Authors:** Du-Yeol Choi, Yonggyun Kim

**Affiliations:** Department of Plant Medicals, College of Life Sciences, Andong National University, Andong, Korea; National Institute of Child Health and Human Development (NICHD), NIH, UNITED STATES

## Abstract

The western flower thrips, *Frankliniella occidentalis*, is one of the most devastating insect pests with explosive reproductive potential. However, its reproductive physiological processes are not well understood. This study reports the ovarian development and associated transcriptomes of *F*. *occidentalis*. Each ovary consisted of four ovarioles, each of which contained a maximum of nine follicles in the vitellarium. The germarium consisted of several dividing cells forming a germ cell cluster, presumably consisting of oocytes and nurse cells. The nurse cells were restricted to the germarium while the subsequent follicles did not possess nurse cells or a nutritive cord, supporting the neo-panoistic ovariole usually found in thysanopteran insects. Oocyte development was completed 72 h after adult emergence (AAE). Transcriptome analysis was performed at mid (36 h AAE) and late (60 h AAE) ovarian developmental stages using RNA sequencing (RNASeq) technology. More than 120 million reads per replication were matched to ≈ 15,000 *F*. *occidentalis* genes. Almost 500 genes were differentially expressed at each of the mid and late ovarian developmental stages. Kyoto Encyclopedia of Genes and Genomes (KEGG) analysis showed that these differentially expressed genes (DEGs) were associated with metabolic pathways along with protein and nucleic acid biosynthesis. In both ovarian developmental stages, vitellogenin, mucin, and chorion genes were highly (> 8-fold) expressed. Endocrine signals associated with ovarian development were further investigated from the DEGs. Insulin and juvenile hormone signals were upregulated only at 36 h AAE, whereas the ecdysteroid signal was highly maintained at 60 h AAE. This study reports the transcriptome associated with the ovarian development of *F*. *occidentalis*, which possesses a neo-panoistic ovariole.

## 1. Introduction

The western flower thrip, *Frankliniella occidentalis* (Pergande) (Thysanoptera: Thripidae), is one of the most devastating insect pests to many horticultural crops, especially those in greenhouses [1]. Both the larval and adult stages cause damage to plants by directly feeding on leaves or flowers. Especially, adults transmit plant viruses including tomato spotted wilt virus (TSWV) [2]. TSWV infection becomes serious and causes massive economic loss in hot pepper production in Korea [3]. This pest, originally native to North America, has spread to more than 60 countries since the late 1970s, including Canada, Australia, the United Kingdom, and far East Asian countries [4].

Various techniques such as chemical insecticides, entomopathogens, and pheromone traps have been applied to control *F*. *occidentalis* without satisfactory efficacy due to the insect’s specific hiding behavior and insecticide resistance [5]. The thrips exhibits arrhenotokous parthenogenesis, with females developing from fertilized eggs and males from unfertilized eggs [6]. A brief immature period less than 10 days along with this various reproductive modes allow the thrips to rapidly build up the field populations during crop cultivating periods and so frequently leads to outbreaks beyond economic injury level [6]. Along with high reproductive potential, this type of mating behavior contributes to a rapid population increase and the development of insecticide resistance [7]. However, the molecular processes underlying reproduction and its regulation in this species remain unclear.

To investigate the physiological processes of the ovarian development of *F*. *occidentalis*, transcriptome analysis is useful for understanding the expressed genes associated with reproduction. A draft genome (415.8 Mb) of *F*. *occidentalis* was sequenced and its 16,859 genes were annotated into different functional categories including chemosensory receptors, detoxification, salivary gland, immunity, and development [8]. This suggests that RNA sequencing (RNASeq) analysis would be highly validated by this genomic information.

To identify the genes associated with ovarian development, this study investigated the ovarian development of *F*. *occidentalis* after adult emergence. After determining the mid and late ovarian developmental stages, transcriptomes were assessed using the NovaSeq 6000 platform. Subsequent differentially expressed gene (DEG) analysis in different developmental stages of female adults predicted the genes associated with ovarian development.

## 2. Materials and methods

### 2.1. Thrip rearing

*F*. *occidentalis* adults were obtained from Bio Utility, Inc. (Andong, Korea) and reared in a laboratory under conditions of 27 ± 1°C constant temperature, a 16:8 h (light:dark) photoperiod, and relative humidity of 60 ± 5%. The insects were reared on sprouted bean seed kernels.

### 2.2. Dissection of ovaries and microscopic observation

Different ages of female western flower thrips were dissected in 1 × phosphate-buffered saline (PBS) under a stereomicroscope at 30× magnification. PBS was prepared with 100 mM phosphate buffer containing 0.7% NaCl (pH 7.4). The ovaries were pulled from the abdominal tip and fixed with 3.7% paraformaldehyde in a wet chamber under darkness at room temperature (RT) for 60 min. After washing three times with 1 × PBS, the cells in the ovarioles were permeabilized with 0.2% Triton X-100 in 1 × PBS at RT for 20 min. The cells were then washed three times and blocked with 5% skim milk (MB cell, Seoul, Korea) in 1 × PBS at RT for 60 min. After washing three times, the cells were incubated with DAPI (4′,6-diamidino-2-phenylindole, 1 mg/mL) diluted 1,000 times in PBS at RT for 2 min for nuclear staining. After washing three times, the ovarian cells were observed under a fluorescence microscope (DM2500, Leica, Wetzlar, Germany) at 200× magnification.

### 2.3. RNA extraction and RNASeq analysis

Total RNAs were extracted from the whole bodies of female *F*. *occidentalis* at different ages (0, 36, and 60 h after adult emergence). Three independent samples were used for three replications at each age. Each sample consisted of 50 females. RNA extraction was performed using Trizol reagent (Invitrogen, Carlsbad, CA, USA) according to the manufacturer’s instructions. An RNA library was generated using TruSeq Stranded Total RNA with Ribo-Zero H/M/R_Gold (Illumina, San Diego, CA, USA). RNA sequencing was performed on the NovaSeq 6000 platform (Illumina) from Macrogen (Seoul, Korea). The RNA sequence was trimmed using CLC Workbench (QIAgen, Hilden, Germany). To calculate relative transcript accumulation, reads per kilobase per million (RPKM) mappable reads of the *F*. *occidentalis* genome (GenBank accession number: GCF_000697945.2) were estimated using CLC Workbench based on a template of Focc_2.1 version with a trimmed sequence of more than 50 bp.

### 2.4. Bioinformatics

DEGs were selected based on a fold change of ≥ 2.0 and a *P*-value of < 0.05 with three biological replicates by comparing RPKM values at 36 h or 60 h after adult emergence (AAE) to those at 0 h AAE. KEGG pathway analysis was performed to test the statistical enrichment of DEGs using the KEGG mapper (https://www.kegg.jp/kegg/) by converting the National Center for Biotechnology Information (NCBI) Gene ID to a KEGG ID through the convert ID tool of KEGG mapper.

### 2.5. RT-qPCR

After RNA extraction, RNA was resuspended in nuclease-free water and quantified using a spectrophotometer (NanoDrop, Thermo Fisher Scientific, Wilmington, DE, USA). RNA (500 ng) was used for cDNA synthesis with RT PreMix (Intron Biotechnology, Seoul, Korea) containing oligo dT primers according to the manufacturer’s instructions. All gene expression levels in this study were determined using a real-time polymerase chain reaction (PCR) machine (Step One Plus Real-Time PCR System, Applied Biosystem, Singapore) under the guidelines of [9]. Real-time PCR was conducted in a reaction volume of 20 μL containing 10 μL of Power SYBR Green PCR Master Mix (Thermo Scientific Korea), 3 μL of cDNA template (200 ng), and each 1 μL (10 pmol) of forward and reverse primers (S1 Table). After initial heat treatment at 95°C for 2 min, qPCR was performed with 40 cycles of denaturation at 95°C for 30 sec, annealing at 53 ~ 55°C for 30 sec, and extension at 72°C for 30 sec. The expression level of elongation factor-1 (*EF-1*, S1 Table) was used as a reference to normalize the target gene expression levels under different treatments. Quantitative analysis was performed using the comparative CT (2^-ΔΔCT^) method [10]. All experiments were independently replicated three times.

### 2.6. Statistical analysis

All the continuous variable data were subjected to a one-way analysis of variance using PROC GLM in the SAS program [11]. Means were compared with Duncan`s multiple range test (DMRT) at type I error = 0.05.

## 3. Results

### 3.1. Ovarian development in *F*. *occidentalis*

**Fig 1:**
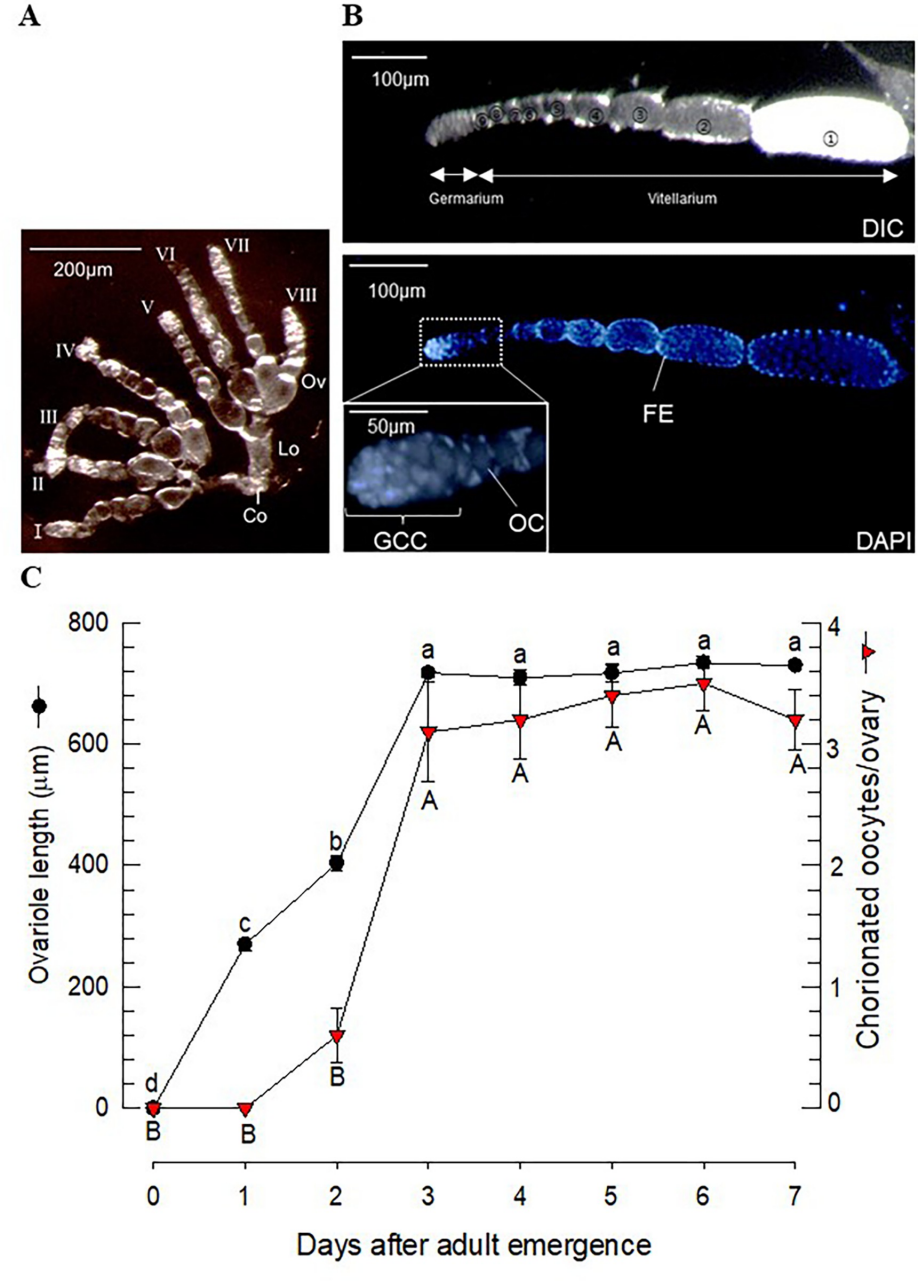
Ovarian development of *F*. *occidentalis*. (A) Internal reproductive organs of 2-day-old females include a pair of ovaries (‘Ov’), 8 ovarioles (‘I-VIII’), a lateral oviduct (‘Lo’), and a common oviduct (‘Co’). (B) Ovariole structure divided into the germarium and the vitellarium. A total of 9 follicles are numbered from proximally to distally in the differential interference contrast (‘DIC’) picture. The white follicle represents chorionated oocytes. In the DAPI picture, each oocyte in the follicle is surrounded by follicular epithelium (‘FE’). In the germarium, the germ cell cluster (‘GCC’) is located near to the oocyte (‘OC’). (C) Ovarian development with female age. The ovariole length represents the germarium and vitellarium. Chorionated oocytes were counted per ovary. Each measurement used individual thrips and was replicated 5 times.

Dissection of 3-day-old female adults showed that a pair of ovaries contained eight ovarioles (Fig 1A). The four ovarioles in each ovary were joined to a lateral oviduct and the two lateral oviducts were combined to the common oviduct. Each ovariole contained a string of follicles and was divided into a germarium and a vitellarium depending upon the presence of matured oocytes (Fig 1B). In the germarium, small cells were closely attached and formed a germ cell cluster. The vitellarium contained nine follicles, each of which was composed of oocytes and follicular epithelium without any nurse cells. The terminal follicles were usually chorionated.

Ovarian development occurred after adult emergence (Fig 1C). The ovariole length began to extend just after adult emergence and reached a maximal size three days after emergence. Chorionated oocytes were visible after two days in some ovarioles. After three days, all the ovarioles had terminal chorionated oocytes, but some of the ovarioles lost them due to oviposition. This ovarian development pattern allowed us to determine three stages: early at 0 h, mid at 36 h, and late at 60 h after adult emergence.

### 3.2. Changes in the transcriptomes of female *F*. *occidentalis* adults during ovarian development

Total RNA was sequenced at three ovarian developmental stages of *F*. *occidentalis*. Each of the nine samples (= 3 stages × 3 replications) was sequenced from 10~13 Gb (Table 1). After trimming, 102~131 million reads in each sample were used to map to the *F*. *occidentalis* genome. With 71~82% mapping rates, the reads in each sample were matched to 14,042~14,521 genes among 16,859 predicted *F*. *occidentalis* genes. All the nine transcriptomes were deposited to GenBank with accession numbers of PRJNA833754.

**Table 1:** Sequencing summary of *F*. *occidentalis* transcripts at different female ages.

Age^1^	N^2^	Total sequences^3^ (bp)	Trimmed reads^4^ (bp)	Mapping^5^ (%)	Matched genes^6^
**0 h**	1	10,320,812,260	102,185,476	71.35	14,298
	2	11,065,530,710	109,558,978	80.16	14,467
	3	13,142,766,804	130,125,490	78.48	14,521
**36 h**	1	13,287,362,040	131,557,128	79.03	14,042
	2	12,494,164,196	123,703,708	82.58	14,154
	3	12,040,868,520	126,733,690	80.40	14,145
**60 h**	1	12,800,190,964	131,932,260	73.82	14,266
	2	13,325,257,038	126,848,436	81.45	14,211
	3	12,811,780,916	126,848,436	73.88	14,119

^1^Age represents the time (h) after adult emergence of females.

^2^ N represents the number of replications. Each replication used 50 females for RNA extraction.

^3^Sequenced by the NovaSeq platform (Illumina, San Diego, CA, USA).

^4^Trimmed by CLC Workbench (QIAgen, Hilden, Germany).

^5^Mapping to the *F*. *occidentalis* genome (GenBank accession number: GCF_000697945.2).

^6^Total number of annotated genes was 16,859.

**Fig 2:**
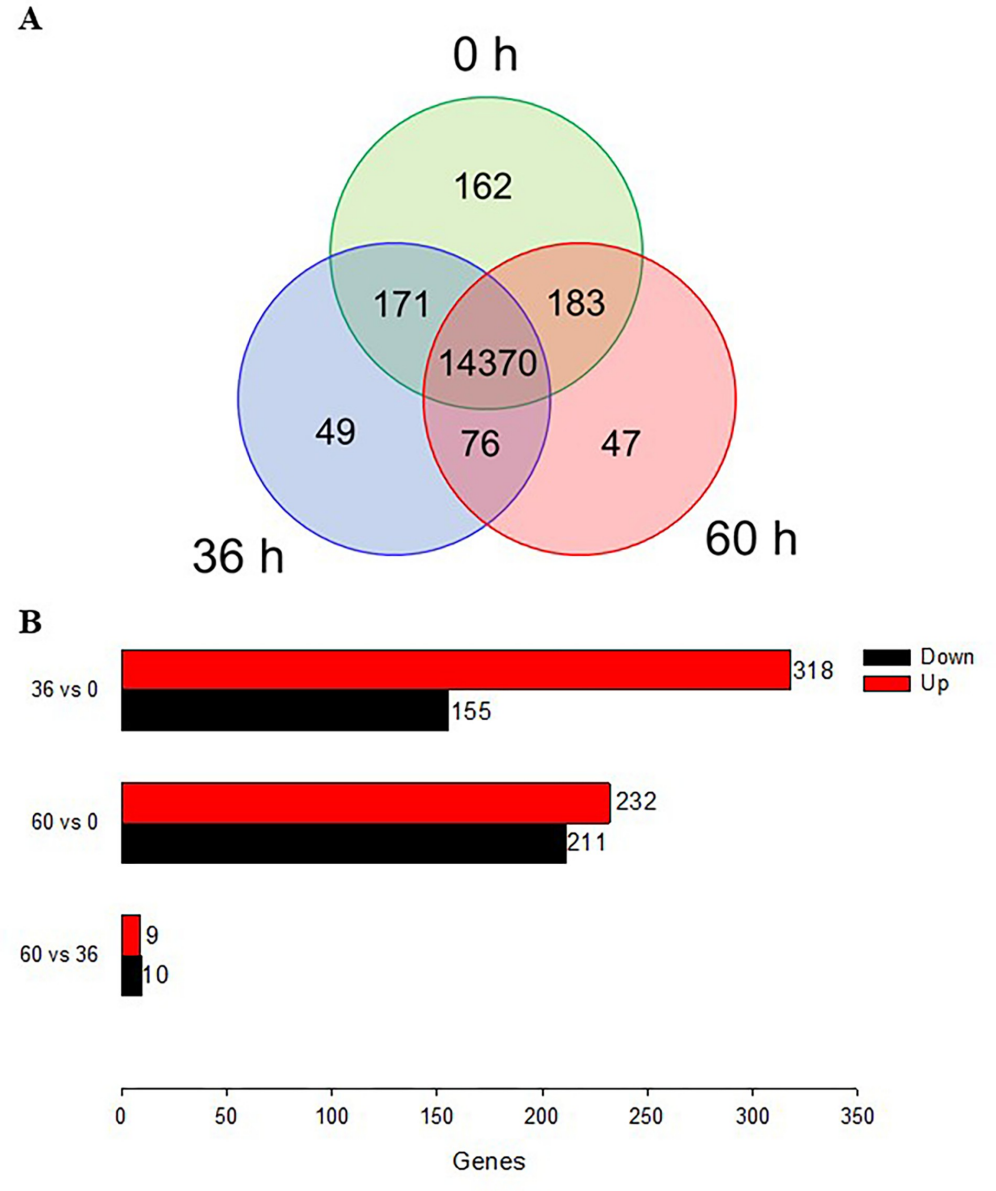
DEG analysis of different ages of *F*. *occidentalis* females. The assessment used genes mapped with RNASeq reads (see Table 1). (A) Venn diagram of total transcripts at 0 h, 36 h, and 60 h after emergence. (B) DEG analysis between two different developmental stages. The threshold was > 2-fold changes in RPKM.

When the transcriptomes of the three developmental stages were compared, they shared more than 95% (= 14,370/15,055) of the transcripts (Fig 2A). Forty-nine unique genes expressed at 36 h AAE were classified according to structure, gene regulation, and cell cycle along with several uncharacterized genes (S2 Table). However, their expression levels were extremely low at 0.0007~0.0468 RPKM. Forty-seven unique genes expressed at 60 h AAE were classified according to structure, protein-processing, and gene regulation along with several uncharacterized genes (S3 Table). Their expression levels were relatively high at 1.0016~1.6941 RPKM. However, this unique gene analysis did not identify genes apparently associated with oogenesis.

To identify *F*. *occidentalis* genes associated with ovarian development, a differentially expressed gene (DEG) analysis was performed (Fig 2B). At 36 h AAE, 473 transcripts showed more than 2-fold increases in gene expression levels compared to those at 0 h AAE. At 60 h AAE, 443 transcripts showed more than 2-fold increases in gene expression levels compared to those at 0 h AAE. Only 19 transcripts showed changes between 36 h and 60 h AAE.

**Fig 3:**
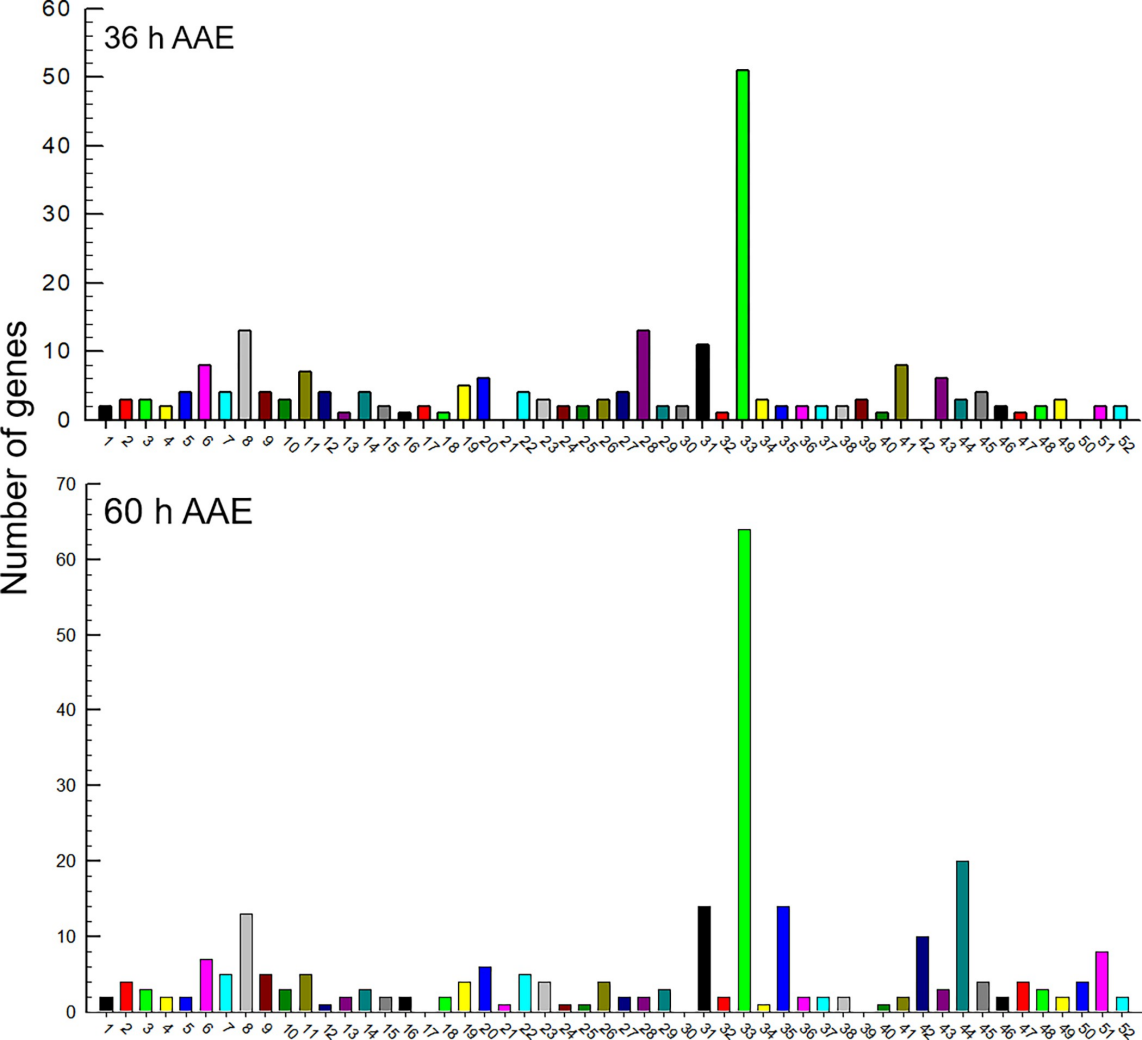
KEGG analysis of the DEGs selected from Fig 2B. The metabolic pathways in the KEGG database were mapped with the DEGs. 36 h AAE represents DEGs between transcripts at 0 h and 36 h after adult emergence and 60 h AAE represents DEGs between transcripts at 0 h and 60 h after adult emergence. KEGG categories include alanine, aspartate and glutamate metabolism (1), amino sugar and nucleotide sugar metabolism (2), apoptosis (3), arachidonic acid metabolism (4), ascorbate and aldarate metabolism (5), autophagy (6), biosynthesis of amino acids (7), biosynthesis of cofactors (8), biosynthesis of nucleotide sugars (9), biosynthesis of unsaturated fatty acids (10), carbon metabolism (11), citrate cycle (12), cysteine and methionine metabolism (13), cytochrome P450 (14), ECM-receptor interaction (15), endocytosis (16), fatty acid biosynthesis (17), fatty acid elongation (18), fatty acid metabolism (19),folate biosynthesis (20), foxO signaling pathway (21), fructose and mannose metabolism (22), glutathione metabolism (23), glycerolipid metabolism (24), glycerophospholipid metabolism (25), glycine, serine and threonine metabolism (26), glycolysis/gluconeogenesis (27), inositol phosphate metabolism (28), longevity regulating pathway (29), lysine degradation (30), lysosome (31), MAPK signaling pathway (32), metabolic pathways (33), mitophagy (34), glycan biosynthesis (35), nicotinate and nicotinamide metabolism (36), nucleotide metabolism (37), oxidative phosphorylation (38), pentose and glucoronate interconversions (39), pentose phosphate pathway (40), phagosome (41), protein export (42), protein processing in endoplasmic reticulum (43), purine metabolism (44), pyruvate metabolism (45), starch and sucrose metabolism (46), steroid biosynthesis (47), thiamine metabolism (48), Toll and Imd signaling pathway (49), various types of N-glycan biosynthesis (50), vitamin B6 metabolism (51), and Wnt signaling pathway (52).

To characterize the DEGs at the mid (36 h AAE) and late (60 h AAE) ovarian developmental stages, their gene functions were predicted using KEGG analysis (Fig 3). Both DEGs were assigned to 52 KEGG functional categories but they did not exactly overlap. The DEGs of each developmental stage were assigned to 49 KEGG categories with three different missing categories. DEGs at 36 h AAE did not include the three categories of #21 (FoxO signaling pathway), #42 (protein export), and #50 (N-glycan biosynthesis), whereas the DEGs at 60 h AAE did not include the three categories of #17 (fatty acid biosynthesis), #30 (lysine degradation), and #39 (pentose and glucuronate interconversion). Among 46 common KEGG categories, most DEGs were classified into the metabolic pathway category (#33) in both developmental stages. The other major (> 10 DEGs) categories, biosynthesis of cofactors (#8) and lysosome (#31), were common in both developmental stages. However, the 36 h AAE samples had more DEGs in the inositol phosphate metabolism category (#28) than the 60 h AAE samples. In contrast, the glycan biosynthesis (#35) and purine metabolism (#44) categories had more DEGs at 60 h AAE than at 36 h AAE. The KEGG analysis suggested the upregulation of metabolic pathways associated with nucleic acid biosynthesis during ovarian development.

### 3.3. Expression profiles of egg proteins during ovarian development

To identify the specific genes associated with oogenesis in *F*. *occidentalis*, we selected genes that were highly expressed more than 8-fold at 36 h AAE or 60 h AAE compared to expression levels at 0 h AAE (Table 2). The selected 99 genes were subdivided into structure, protein processing, lipid metabolism, gene regulation, and others. The structural protein category included typical egg proteins such as vitellogenin, chorion protein, mucin, and yellow melanization protein. In contrast, the highly suppressed genes at these stages included 37 genes (S4 Table). Especially, larval cuticular protein genes were included in the suppressed gene category.

**Table 2:** Highly (> 8-fold) expressed genes at mid (36 h after adult emergence) and late (60 h) ovarian development stages compared to expression levels at the early (0 h) developmental stage in female *F*. *occidentalis* adults.

Category (99)	NCBI Gene ID	GenBank accession	Annotation	36 h		60 h	
				RPKM	Log_2_ Fc	RPKM	Log_2_ Fc
**Structure (18)**	LOC113214498	XM_026433864.1	vitellogenin	2.69	4.13	2.58	3.77
	LOC113214411	XM_026433762.1	vitellogenin-2	2.60	4.44	2.58	4.38
	LOC113202497	XM_026416747.1	vitellogenin-1	2.60	3.35	2.64	3.51
	LOC113204471	XM_026419639.1	vitellogenin-1	2.75	4.05	2.73	4.01
	LOC113212925	XM_026431801.1	vitellogenin-1	1.68	3.71	1.63	3.54
	LOC113205436	XM_026421068.1	flocculation protein	1.21	3.42	1.07	2.94
	LOC113214189	XM_026433479.1	endoglucanase-5	0.95	2.53	1.17	3.27
	LOC113211573	XM_026429977.1	chorion class A protein	2.70	5.12	2.56	4.67
	LOC113202531	XM_026416797.1	myosin heavy chain	1.70	4.05	1.54	3.50
	LOC113213533	XM_026432640.1	mucin-5AC	1.87	4.25	1.82	4.06
	LOC113217829	XM_026437896.1	filaggrin	1.04	3.33	0.95	3.01
	LOC113206591	XM_026422740.1	filaggrin-2	1.19	3.02	1.15	2.89
	LOC113217875	XM_026437953.1	keratin-associated protein	1.67	4.55	1.58	4.27
	LOC113217915	XM_026438006.1	keratin-associated protein	1.64	4.84	1.56	4.56
	LOC113218104	XM_026438307.1	protein yellow	2.70	5.29	2.62	5.00
	LOC113206471	XM_026422568.1	nacrein	2.34	3.94	2.41	4.18
	LOC113214093	XM_026433360.1	sperm acrosome-associated protein 5	2.36	5.65	2.50	6.12
	LOC113217267	XM_026437086.1	venom allergen	1.96	5.17	2.02	5.39
**Protein processing (23)**	LOC113216008	XM_026435682.1	transmembrane protein	2.16	5.40	2.05	5.03
	LOC113214127	XM_026433402.1	PE-PGRS family protein	2.08	3.50	1.83	2.67
	LOC113209730	XM_026427399.1	trypsin	1.69	3.73	1.39	2.72
	LOC113204180	XM_026419243.1	trypsin	1.95	3.66	1.77	3.07
	LOC113203267	XM_026417851.1	cathepsin L1	3.28	3.01	3.17	2.66
	LOC113205904	XM_026421697.1	cathepsin L1	1.57	2.81	1.71	3.28
	LOC113212259	XM_026430877.1	carboxypeptidase B	1.08	3.16	1.04	3.03
	LOC113202896	XM_026417339.1	transmembrane protease serine 9	1.75	3.26	1.53	2.54
	LOC113206464	XM_026422562.1	transmembrane protease serine 9	1.97	4.45	1.89	4.20
	LOC113214490	XM_026433856.1	probable pectin lyase D	1.93	4.93	2.09	5.45
	LOC113207878	XM_026424617.1	probable pectin lyase B	2.13	3.87	2.02	3.50
	LOC113216785	XM_026436611.1	probable pectin lyase B	2.93	5.59	3.11	6.17
	LOC113216787	XM_026436612.1	probable pectin lyase B	1.93	4.76	1.99	4.95
	LOC113211651	XM_026430098.1	pectin lyase	1.27	3.11	1.11	2.57
	LOC113203564	XM_026418330.1	pectin lyase	1.16	2.79	1.24	3.05
	LOC113215606	XM_026435246.1	lysozyme C milk isozyme	1.28	3.61	1.43	4.11
	LOC113217852	XM_026437921.1	polyhomeotic-proximal chromatin protein	1.30	3.46	1.19	3.09
	LOC113208437	XM_026425417.1	transcriptional regulatory protein AlgP	2.03	3.32	2.19	3.85
	LOC113217886	XM_026437969.1	cyclin-dependent kinase inhibitor	1.38	4.24	1.41	4.36
	LOC113210725	XM_026428834.1	PE-PGRS family protein	2.42	3.86	2.14	2.93
	LOC113212288	XM_026430917.1	protein rtoA	0.80	2.05	1.24	3.52
	LOC113207332	XM_026423852.1	hornerin	1.96	3.14	1.98	3.21
	LOC113206614	XM_026422771.1	hornerin	2.12	3.84	2.10	3.75
**Lipid metabolism (6)**	LOC113208001	XM_026424805.1	clavesin-1	1.31	3.63	1.17	3.15
	LOC113213966	XM_026433193.1	pancreatic triacylglycerol lipase	1.33	3.17	1.33	3.16
	LOC113215706	XM_026435368.1	low-density lipoprotein receptor	1.20	3.38	1.06	2.94
	LOC113214129	XM_026433404.1	lipase member K	1.02	3.03	1.05	3.14
	LOC113202163	XM_026416266.1	acyl-CoA Delta (11) desaturase	1.80	3.82	1.45	2.66
	LOC113202156	XM_026416257.1	phospholipase A1	1.73	3.00	1.60	2.58
**Gene regulation (3)**	LOC113211421	XM_026429785.1	serine-rich adhesin for platelets	1.44	4.15	1.41	4.06
	LOC113217849	XM_026437920.1	serine-rich adhesin for platelets	1.58	4.98	1.50	4.73
	LOC113210704	XM_026428809.1	regucalcin	1.50	3.58	1.48	3.51
**Others (49)**	LOC113204174	XM_026419235.1	histidine-rich glycoprotein	2.05	4.91	1.86	4.27
	LOC113216845	XM_026436674.1	histidine-rich glycoprotein	3.02	4.16	2.98	4.04
	LOC113217182	XM_026437013.1	non-classical arabinogalactan protein	1.11	3.55	0.97	3.06
	LOC113202260	XM_026416395.1	neurofilament medium polypeptide	1.58	4.19	1.42	3.65
	LOC113202298	XM_026416451.1	proline-rich protein 2	2.10	3.76	2.03	3.55
	LOC113217885	XM_026437968.1	uncharacterized	2.53	4.89	2.45	4.62
	LOC113217883	XM_026437966.1	uncharacterized	1.33	4.33	1.25	4.07
	LOC113217830	XM_026437897.1	uncharacterized	1.53	4.33	1.49	4.18
	LOC113217828	XM_026437895.1	uncharacterized	1.08	3.32	0.97	2.97
	LOC113217577	XM_026437533.1	uncharacterized	1.60	4.62	1.93	5.71
	LOC113217326	XM_026437181.1	uncharacterized	1.67	3.66	1.69	3.74
	LOC113216895	XM_026436728.1	uncharacterized	1.13	2.47	1.30	3.02
	LOC113216056	XM_026435756.1	uncharacterized	2.67	4.35	2.69	4.42
	LOC113216047	XM_026435745.1	uncharacterized	1.47	3.39	1.21	2.53
	LOC113214206	XM_026433496.1	uncharacterized	1.98	3.53	1.92	3.32
	LOC113213753	XM_026432913.1	uncharacterized	1.79	3.62	1.66	3.21
	LOC113212826	XM_026431653.1	uncharacterized	1.70	4.11	1.53	3.56
	LOC113212825	XM_026431652.1	uncharacterized	1.50	3.68	1.34	3.15
	LOC113212293	XM_026430924.1	uncharacterized	0.68	1.85	1.07	3.14
	LOC113212290	XM_026430919.1	uncharacterized	0.87	2.52	1.28	3.89
	LOC113212282	XM_026430908.1	uncharacterized	2.63	3.06	2.60	2.98
	LOC113211882	XM_026430401.1	uncharacterized	1.77	4.33	1.58	3.72
	LOC113211714	XM_026430181.1	uncharacterized	2.37	4.68	2.31	4.48
	LOC113211400	XM_026429765.1	uncharacterized	1.54	4.46	1.93	5.75
	LOC113211399	XM_026429764.1	uncharacterized	1.69	3.72	2.00	4.74
	LOC113211078	XM_026429332.1	uncharacterized	2.19	4.85	2.24	4.99
	LOC113210730	XM_026428840.1	uncharacterized	1.99	3.31	1.85	2.87
	LOC113210583	XM_026428652.1	uncharacterized	1.74	3.00	1.70	2.86
	LOC113210342	XM_026428298.1	uncharacterized	1.79	4.37	1.97	4.99
	LOC113208836	XM_026426044.1	uncharacterized	1.39	4.26	1.29	3.93
	LOC113208574	XM_026425626.1	uncharacterized	1.32	3.16	1.28	3.04
	LOC113208471	XM_026425481.1	uncharacterized	1.62	3.41	1.54	3.13
	LOC113208012	XM_026424840.1	uncharacterized	2.21	3.98	1.92	3.01
	LOC113206973	XM_026423287.1	uncharacterized	2.18	3.50	1.90	2.57
	LOC113206617	XM_026422774.1	uncharacterized	1.49	3.41	1.28	2.70
	LOC113205710	XM_026421427.1	uncharacterized	2.21	5.77	2.32	6.12
	LOC113205709	XM_026421426.1	uncharacterized	2.13	5.75	2.23	6.09
	LOC113205708	XM_026421425.1	uncharacterized	2.56	5.69	2.73	6.24
	LOC113205435	XM_026421067.1	uncharacterized	1.49	3.43	1.28	2.72
	LOC113205325	XM_026420899.1	uncharacterized	2.30	4.79	2.29	4.77
	LOC113205123	XM_026420596.1	uncharacterized	1.29	3.62	0.84	2.11
	LOC113204714	XM_026419980.1	uncharacterized	1.79	3.52	1.67	3.14
	LOC113204504	XM_026419693.1	uncharacterized	1.43	3.32	1.45	3.39
	LOC113204057	XM_026419056.1	uncharacterized	2.05	3.55	2.03	3.51
	LOC113203620	XM_026418390.1	uncharacterized	1.57	4.42	1.88	5.46
	LOC113203560	XM_026418318.1	uncharacterized	1.52	3.65	1.52	3.66
	LOC113203559	XM_026418317.1	uncharacterized	1.33	3.18	1.25	2.89
	LOC113203548	XM_026418307.1	uncharacterized	2.25	4.83	2.29	4.96
	LOC113202588	XM_026416887.1	uncharacterized	2.39	4.90	2.25	4.43

**Fig 4:**
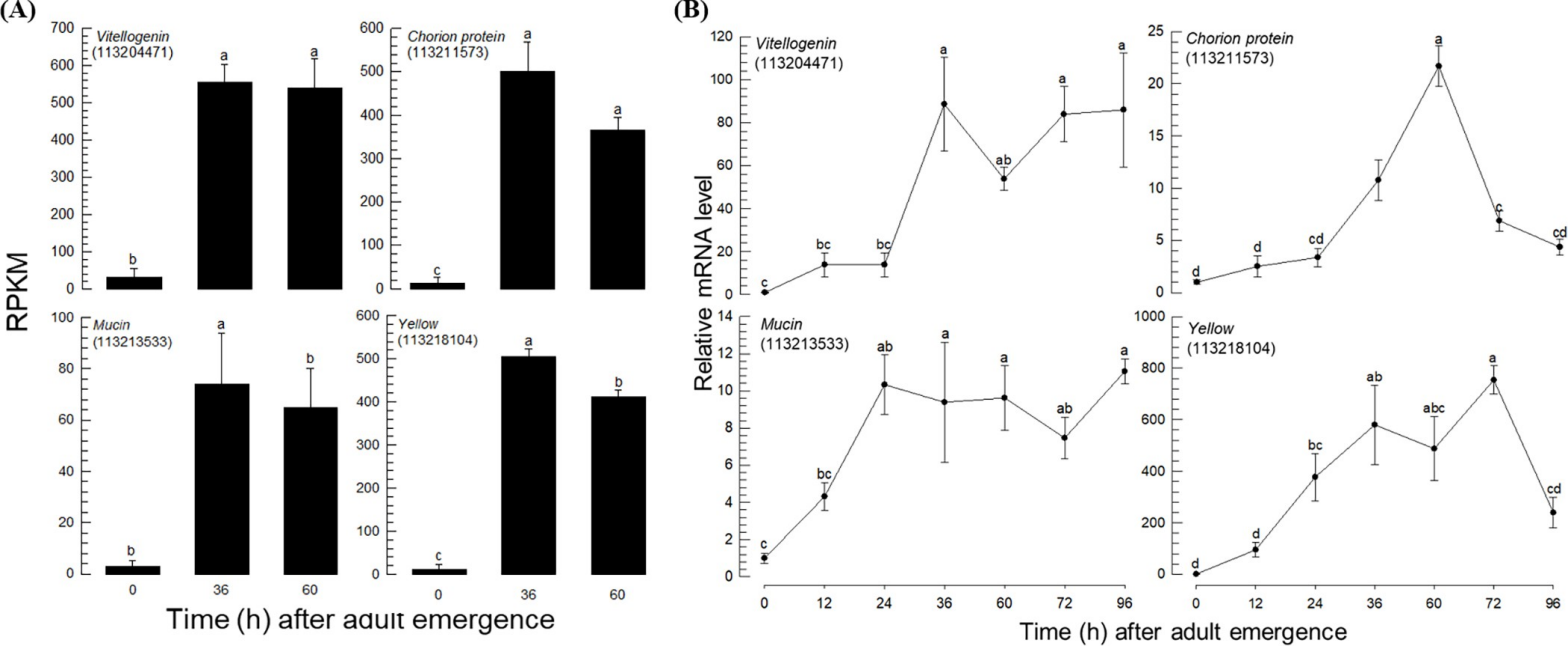
Expression analysis of the selected egg proteins during *F*. *occidentalis* ovarian development. The NCBI Gene ID is in each parenthesis. (A) RNASeq analysis. (B) RT-qPCR analysis. Each measurement was replicated 3 times. The figures in parentheses indicate LOCs, which are the NCBI gene IDs. Different letters above the standard deviation bars represent significant differences among the means at type I error = 0.05 (DMRT test)].

The expression patterns of representative egg proteins during adult development were further analyzed (Fig 4). As expected, RNASeq analysis found that *vitellogenin*, *chorion protein*, *mucin*, and *yellow* genes were highly expressed in the mid and late ovarian developmental stages (Fig 4A). However, there was little or no difference in the expression levels between the two developmental stages (36 h AAE and 60 h AAE). These transcript level profiles were further assessed by RT-qPCR with additional development stages (Fig 4B). The expression levels measured by RT-qPCR were mostly consistent with the expression profiles measured by RNASeq. However, RT-qPCR analysis indicated that the *mucin* and *yellow* genes were induced earlier than 36 h AAE. It also showed their expression patterns in late ovarian development after 60 h AAE, in which *vitellogenin* and *mucin* maintained the induced levels, whereas the expression levels of *chorion protein* and *yellow* significantly decreased.

### 3.4. Expression profiles of genes associated with endocrine signals during ovarian development

**Fig 5:**
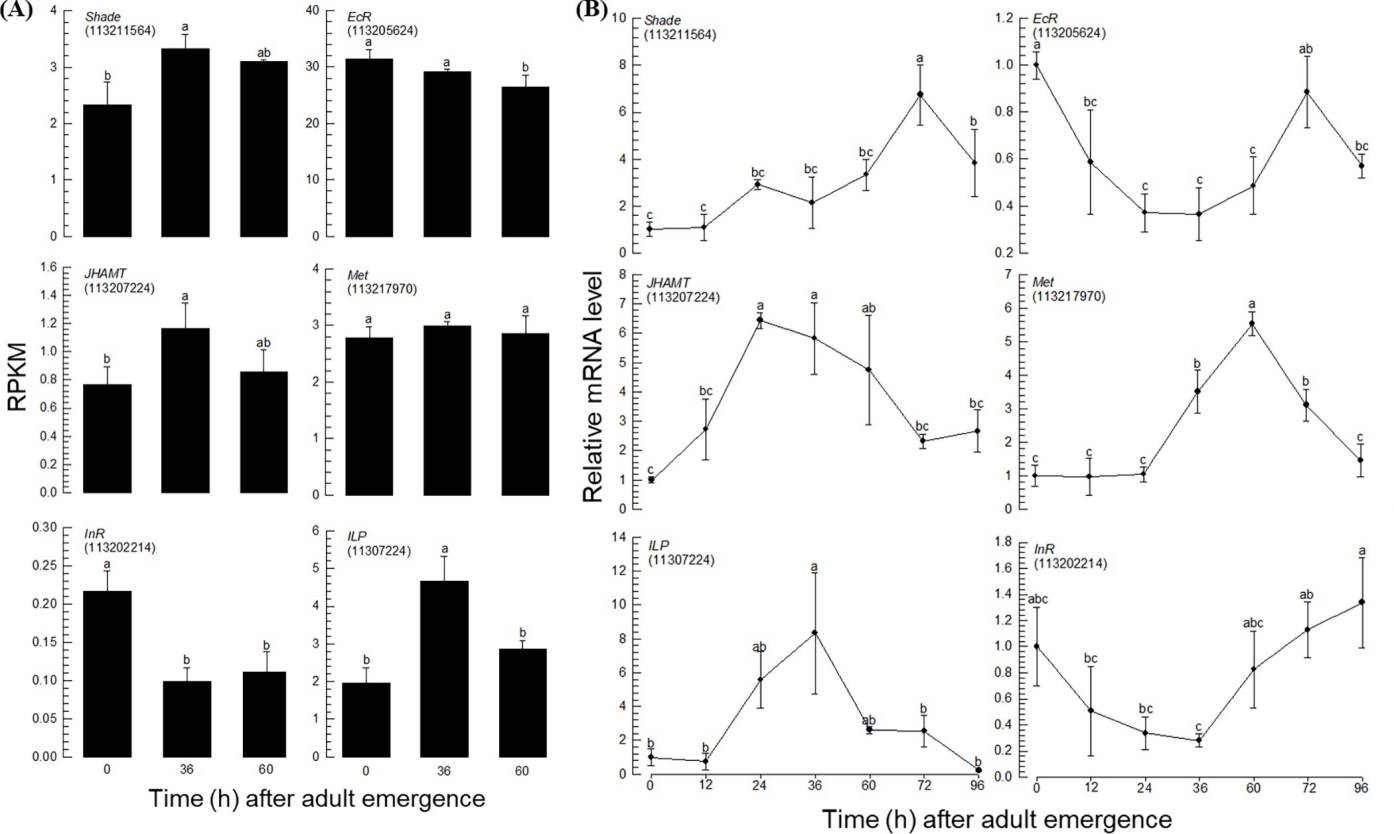
Expression analysis of the selected endocrine signal genes during *F*. *occidentalis* ovarian development. The NCBI Gene ID is in each parenthesis. (A) RNASeq analysis. (B) RT-qPCR analysis. Each measurement was replicated 3 times. The figures in parentheses indicate LOCs, which are the NCBI gene IDs. Different letters above the standard deviation bars represent significant differences among the means at type I error = 0.05 (DMRT test).

Juvenile hormone (JH), ecdysteroid, and insulin-like peptide (ILP) are well-known endocrine mediators during insect reproduction [12]. Genes associated with these endocrine signals were selected from the transcriptomes (Table 3). JH acid methyltransferase (JHAMT), JH esterase/JH epoxide hydrolase, and Met involved in JH synthesis, JH degradation, and the JH receptor, respectively, were identified. The RNASeq analysis showed that JHAMT was highly upregulated at 36 h AAE, whereas Met expression levels did not change during adult development (Fig 5A). As ecdysteroid signaling genes, *Shade* and *EcR* were found in the adult transcriptomes. The expression levels of *Shade* were upregulated at mid and late ovarian development, during which *EcR* expression was slightly decreased. Insulin-like peptides and receptors were included in the adult transcriptomes. *ILP* was highly upregulated at 36 h AAE, whereas *InR* expression levels decreased during adult development. The transcriptome profiles related to endocrine signals were further supported by RT-qPCR analysis (Fig 5B). The RT-qPCR analysis assessed at several time points after adult emergence showed the upregulation of these endocrine signals during ovarian development. Especially, JH and insulin signals were more upregulated at 36 h AAE than at 60 h AAE, whereas the ecdysteroid signal was more upregulated at the late developmental stage.

**Table 3:** DEGs associated with endocrine signals in female *F*. *occidentalis* at mid (36 h after adult emergence) and late ovarian development stages compared to the early (0 h) stage.

Category	NCBI Gene ID	GenBank Accession	Annotation	36 h		60 h	
				RPKM	Log_2_Fc	RPKM	Log_2_Fc
**Juvenile hromone (JH)**	LOC113205672	XM_026421382.1	JH esterase	2.78	0.11	2.08	-0.31
	LOC113206119	XM_026422046.1	JH epoxide hydrolase	48.44	2.32	37.15	1.94
	LOC113206791	XM_026423048.1	JH esterase	6.75	1.66	5.04	1.24
	LOC113207106	XM_026423497.1	JH esterase	0.61	-1.74	0.48	-2.09
	LOC113207121	XM_026423514.1	JH esterase	0.17	-0.38	0.11	-1.06
	LOC113208804	XM_026425987.1	JH esterase	0.92	-1.16	1.17	-0.81
	LOC113209084	XM_026426427.1	JH esterase	0.77	1.18	0.59	0.81
	LOC113211838	XM_026430351.1	JH esterase	8.02	4.27	8.17	4.30
	LOC113215323	XM_026434935.1	JH-suppressible protein	0.72	-3.37	0.23	-5.01
	LOC113202122	XM_026416212.1	JH esterase	3.67	-0.28	3.48	-0.36
	LOC113202626	XM_026416948.1	JH acid methyltransferase	1.17	0.61	0.57	-0.43
	LOC113202308	XM_026416465.1	JH esterase	0.95	-0.30	0.77	-0.60
	LOC113217970	XM_026416465.2	Methoprene tolerance	2.99	0.10	2.60	-0.10
**Ecdysteroid**	LOC113205624	XM_026421313.1	Shade	3.32	0.51	2.96	0.35
	LOC113207454	XM_026424033.1	ecdysone receptor	0.21	-1.80	0.18	-2.04
	LOC113211564	XM_026429966.1	ecdysone receptor	29.17	-0.11	23.39	-0.42
	LOC113211835	XM_026430346.1	zinc finger protein on ecdysone	4.31	0.22	4.09	0.14
	LOC113211939	XM_026430486.1	ecdysone-induced protein	0.77	-1.43	0.42	-2.33
	LOC113214771	XM_026434236.1	protein ecdysoneless	6.68	0.23	6.47	0.18
	LOC113216508	XM_026436255.1	protein ecdysoneless	1.64	0.02	1.64	0.02
	LOC113216945	XM_026436781.1	ecdysone-induced protein 74EF	1.72	-1.37	1.74	-1.35
**Insulin**	LOC113202214	XM_026416323.1	IGF1 receptor	0.10	-1.14	0.10	-1.17
	LOC113206117	XM_026422043.1	insulin-like receptor	5.22	-0.27	4.67	-0.43
	LOC113207224	XM_026423730.1	insulin-like peptide	4.67	1.25	2.36	0.27
	LOC113209425	XM_026426931.1	IGF2-BP	17.36	0.50	13.70	0.16
	LOC113210134	XM_026427972.1	insulin-degrading enzyme	8.92	0.03	8.04	-0.12
	LOC113211136	XM_026429429.1	IGF-BP complex acid labile subunit	68.21	0.31	48.47	-0.19
	LOC113211621	XM_026430058.1	IGF-BP complex acid labile subunit	2.53	0.47	1.85	0.02
	LOC113211864	XM_026430384.1	IGF-BP complex acid labile subunit	9.49	-0.57	6.76	-1.06
	LOC113212596	XM_026431366.1	IGF-BP complex acid labile subunit	2.06	-0.49	1.56	-0.89
	LOC113212620	XM_026431414.1	IGF-BP complex acid labile subunit	0.69	-0.70	0.49	-1.21
	LOC113215479	XM_026435105.1	IGF-BP7	0.09	-0.75	0.06	-1.34

## 4. Discussion

Various reproductive modes are observed in Thysanoptera. In *F*. *occidentalis*, a female produces progeny in bisexual or asexual arrhenotokous reproduction [13]. In arrhenotokous reproduction in the situation of few males (e.g., overwintering population), virgin females produce only male offspring [6]. When their sons are sexually mature, the females undergo bisexual reproduction with their sons and produce female-biased offspring. All thrips including *F*. *occidentalis* exhibit oviparous reproduction, in which females in either the asexual or bisexual mode undergo oogenesis in their ovaries. However, little is known about ovarian development in *F*. *occidentalis*. The current study analyzed the internal reproductive organ structure of *F*. *occidentalis* to assess the developing oocytes. Based on the temporal developmental pattern, RNASeq analysis was performed at early, mid, and late stages to determine the specific genes associated with ovarian development.

The ovaries and associated internal reproductive organs of *F*. *occidentalis* were observed. Eight ovarioles from a pair of ovaries contained follicles, the end of which contained chorionated oocytes. Each follicle was composed of oocytes and follicular epithelium. The distal germarium contained a germ cell cluster, presumably consisting of interconnected nurse cells and oocytes. The ovarioles of insects are categorized into panoistic and meroistic types, in which the latter type is subdivided into polytrophic and telotrophic groups [14]. The panoistic type is considered to be the most ancestral because of its deficiency in transforming oogonia into nurse cells [15]. The polytrophic meroistic ovary has evolved from the panoistic ovary through the differentiation of nurse cells, and finally, the telotrophic meroistic type is derived from the polytrophic meroistic type by the restriction of nurse cells to the germarium. A deviation from the typical ovariole types is observed in Thysanoptera, in which the germ cell cluster is formed as seen in the ovary of the terebrantian thrip, *Purthenothrips drucenae* [16], suggesting that the panoistic follicles resulted from the secondary loss of nurse cells from the germ cell cluster. Stys et al. [14] called this type of ovary “neo-panoistic.” Thus, the thysanopteran ovary provided new insight into the evolution of insect ovaries. Later, tubuliferan thrips also showed germ cell clusters, indicating that the neo-panoistic ovariole-type prevailed in Thysanoptera [17]. This was supported by our current study using *F*. *occidentalis* ovarioles, which did not have nurse cells in the follicles, while a germ cell cluster was found in the germarium.

The ovaries of *F*. *occidentalis* grew just after adult emergence. During this period, the ovariole size increased along with oocyte development, and the final follicles in each ovariole had chorionated oocytes. Oogenesis is a sequential process consisting of previtellogenic development, vitellogenesis, and choriogenesis [18]. Previtellogenic development occurs in the germarium at the distal part of each ovariole and forms oocytes from the oogonial stem cells by mitosis and meiosis. Vitellogenesis is the process of accumulating vitellogenin (Vg) and other biomaterials into growing oocytes. After oocytes are fully grown, they are coated with chorion proteins secreted from the follicular epithelium to become eggs at the proximal part of the ovarioles. These eggs are then ovulated to the oviducts and fertilized just before oviposition. This ovarian developmental scenario suggests a sequence of oogenesis events in the neo-panoistic ovariole of *F*. *occidentalis*. First, primary oocytes may be produced from germ cell clusters in the germarium. Second, the oocytes grow in size by accumulating Vg during vitellogenesis. Finally, the follicular epithelium forms the chorion of the fully grown oocytes during choriogenesis. Our transcriptome analysis supported the oogenesis processes by providing expression profiles of *Vg*, *chorion proteins*, *mucin*, and *yellow* genes during oogenesis.

RNASeq analysis used the NovaSeq platform, which sequenced more than 100 million reads per sample and resulted in more than 80% gene mapping rates. The first draft genome of *F*. *occidentalis* was reported and 16,859 genes were annotated [8]. Our RNASeq data from three ovarian developmental stages were mapped to 15,083 genes. Most of the mapped genes were shared among the three ovarian developmental stages. DEG analysis identified 473 and 443 DEGs in the mid and late stages, respectively. These DEGs were associated with metabolic pathways, especially related to nucleic acid biosynthesis and cofactor and amino acid biosynthetic pathways. The findings suggest that ovarian development in *F*. *occidentalis* requires a massive supply of raw materials such as nucleic acids and proteins.

Egg proteins were selected from the transcriptomes of the ovarian developmental stages. These genes represented highly expressed genes because they were increased more than 8-fold compared to the early ovarian developmental stage. The genes included *mucin* and *yellow* in addition to the well-known *Vg* and *chorion protein* genes. Mucins are high molecular and heavily glycosylated proteins with tandem repeats of identical or highly similar sequences rich in Ser, Thr, and Pro [19]. In insects, intestinal mucin is a major protein in the midgut peritrophic membrane, which facilitates the digestive process as well as protects the gut epithelium from microbial infections [20]. Salivary gland mucin might modulate the lubrication of insect mouthparts or defend plant attachment by inducing plant cell death through the formation of salivary sheaths [21, 22]. A specific mucin protein is known to be associated with the formation of eggshells in *Nilaparvapa lugens* and *Spodoptera exigua* [23, 24]. In our current study, the *mucin* gene highly expressed during *F*. *occidentalis* ovarian development suggests its function in chorion formation. Yellow and related major royal jelly protein (MRJP)-like proteins are widely found in insect genomes and these genes are classified into ten clades including Yellow-b, -c, -d/e3, -e, -f, -g/g2, -h, -y, -x and MRJP-like protein [25]. In the *Aedes albopictus* mosquito, Yellow-g and Yellow-g2 are localized in the exochorion and outer endochorion, where they mediate darkening processes to physically strengthen the chorions [26]. Thus, the high expression of the *yellow* gene during *F*. *occidentalis* ovarian development suggests its function in chorion formation.

Transcriptome analysis also showed the expression profiles of genes associated with endocrine signals during *F*. *occidentalis* ovarian development. In insects, different endocrine signals are associated with ovarian development. JH is a sesquiterpenoid that mediates a status quo effect during the immature stage to prevent precocious metamorphosis. However, in adults, it stimulates ovarian development as a gonadotropin in various insects [27]. JH directly stimulates Vg biosynthesis in some insects and facilitates Vg uptake by growing oocytes by inducing follicular patency [28]. In mosquito females, 20-hydroxyecdysone acts as a gonadotropin [29]. ILPs are known to mediate ovarian development by stimulating oogonial proliferation to produce oocytes in the stem cell niche located in the germarium of the distal ovariole [30]. In *F*. *occidentalis*, JH and ecdysteroid play crucial roles in mediating metamorphosis. Krüppel homolog 1 (*Kr-h1*) and Broad (*Br*) are transcription factors leading to larval and pupal characteristics under JH and ecdysteroid hormones, respectively [31, 32]. In *F*. *occidentalis*, Kr-h1 mRNA levels were high in the embryonic stage, remained at a moderate level in the larval and prepupal stages, and were low in the pupal stage. In contrast, *Br* mRNA levels were moderate in the embryonic stage and high at the larva-pupa transition stage. Except for *Br* expression in the embryonic stage, these two gene expression patterns followed the corresponding profiles of holometamorphic insects [33]. Furthermore, the adult specifier, *E93*, expression increased during immature development and its inhibition prevented adult metamorphosis [34]. However, little is known about JH and ecdysteroid mediation in oogenesis in *F*. *occidentalis*. Our current transcriptome analysis during ovarian development suggests that these endocrine signals play crucial roles in mediating *F*. *occidentalis* oogenesis based on their expression profiles. Increases in *ILP* and *JHAMT* expression at the mid ovarian developmental stage suggest their mediation of previtellogenesis by providing new oocytes from stem cells and vitellogenesis by stimulating Vg synthesis and uptake. Maintaining high levels of *Shade* expression suggest a high level of ecdysteroid during ovarian development, which may stimulate metabolic pathways, especially protein and nucleic acid biosynthesis, in addition to stimulating Vg synthesis with the cooperation of JH.

This study reports the comparative transcriptomes of *F*. *occidentalis* during different stages of ovarian development. Although the transcriptome analyses do not completely represent the protein expression profiles, they gave us valuable insights on the thrips reproduction. The results suggest an increase in metabolic pathways along with protein and nucleic acid biosynthesis. The high upregulation of egg proteins such as Vg, chorion protein, and sclerotizing agents during choriogenesis was also found. Finally, JH, ecdysteroid, and insulin signals may play crucial roles in mediating *F*. *occidentalis* oogenesis. A recent study showed that prostaglandin mediates oocyte devilment in early and late stages in addition to the endocrine signals [35]. This suggests the oogenesis of *F*. *occidentalis* would be a model system for an integrative analysis of endocrine signals mediating different reproductive processes of previtellogenesis, vitellogenesis, and choriogenesis.

## Supporting information

S1 Table Primers used in this study. (DOCX)

S2 Table Annotation of 53 genes expressed only at 36 h after adult emergence (AAE) compared to expression levels at the early (0 h AAE) developmental stage in female *F. occidentalis* adults. (DOCX)

S3 Table Annotation of 68 genes expressed only at 60 h after adult emergence (AAE) compared to expression levels at the early (0 h AAE) developmental stage in female *F. occidentalis* adults. (DOCX)

S4 Table Highly (> 8-fold) suppressed genes at mid (36 h after adult emergence) and late (60 h after adult emergence) ovarian development stages compared to expression levels in the early (0 h after adult emergence) developmental stage in female *F. occidentalis* adults. (DOCX)
