# Supplementary material for: Transcriptome analysis of female western flower thrips, Frankliniella occidentalis, exhibiting neo-panoistic ovarian development
Source: PLoS One. 2022 Aug 1;17(8):e0272399. doi: 10.1371/journal.pone.0272399 (PMC9342723; doi:10.1371/journal.pone.0272399)
Supplement: S1 Table — (DOCX) [file pone.0272399.s001.docx]

**Table S1.** Primers used in this study

| Gene | Direction (5`->3`) | Size (bp) | Annealing (^o^C) |
| --- | --- | --- | --- |
| *JHAMT* | AAATCGCGAAAAGACCTACA | 222 | 55 |
|  | TTTCTTGCGTGAACTACCAG |  |  |
| *Met* | ATATTACCTGAGGGGTGTCT | 256 |  |
|  | GAATTGTTGTTTGGTGCAGT |  |  |
| *Shade* | TACGGAATGCCCCTTATTTG | 256 |  |
|  | CCACAGCCAAATGGTAAAAC |  |  |
| *Ecr* | GAACCATACAACCGATCAGA | 277 |  |
|  | TGAGGACTGCAAGAAGTTTT |  |  |
| *EF1* | TCAAGGAACTGCGTCGTGGAT | 110 |  |
|  | ACAGGGGTGTAGCCGTTAGAG |  |  |
| *Mucin* | AAACCTTCGATAAGTCCACC | 276 |  |
|  | TGACCTTCATGGTAAACTCG |  |  |
| *Yellow* | AGTTCCTGTTCTTCCGTTTC | 229 |  |
|  | ATCACGTTGATGACCTGGTA |  |  |
| *Vitellogenin* | AGGAGATGAACCTCGCTTA | 334 | 53 |
|  | GGAAGTTAGCCTCAACCTG |  |  |
| *Chorion protein* | GCTCTTCGAGATCGCTTC | 334 |  |
|  | CTAGCAGTCCCAGCTCTT |  |  |
