# Supplementary material for: Transcriptome analysis of female western flower thrips, Frankliniella occidentalis, exhibiting neo-panoistic ovarian development
Source: PLoS One. 2022 Aug 1;17(8):e0272399. doi: 10.1371/journal.pone.0272399 (PMC9342723; doi:10.1371/journal.pone.0272399)
Supplement: S2 Table — (DOCX) [file pone.0272399.s002.docx]

**Table S2.** Annotation of 53 genes expressed only at 36 h after adult emergence (AAE) compared to expression levels at the early (0 h AAE) developmental stage in female *F. occidentalis* adults

| Category  (49)^1^ | NCBI  Gene ID | GenBank  accession | Annotation | RPKM |
| --- | --- | --- | --- | --- |
| Structural  proteins (8) | LOC113211259 | XM_026429581.1 | skin secretory protein xP2 | 1.0234±0.0405 |
|  | LOC113203687 | XM_026418482.1 | serine/arginine repetitive matrix protein | 1.0038±0.0066 |
|  | LOC113211343 | XM_026429693.1 | vegetative cell wall protein gp1 | 1.0047±0.0082 |
|  | LOC113216613 | XM_026436357.1 | Wiskott-Aldrich syndrome protein family member 2 | 1.0083±0.0144 |
|  | LOC113216420 | XM_026436172.1 | atherin | 1.0083±0.0144 |
|  | LOC113211370 | XM_026429728.1 | tektin-4 | 1.0091±0.0158 |
|  | LOC113203068 | XM_026417555.1 | endocuticle structural glycoprotein SgAbd-8 | 1.0129±0.0223 |
|  | LOC113213307 | XM_026432331.1 | predicted GPI-anchored protein 58 | 1.0163±0.0282 |
| Gene regulation (14) | LOC113205865 | XM_026421642.1 | piggyBac transposable element-derived protein 4 | 1.0157±0.0157 |
|  | LOC113213614 | XM_026432731.1 | translation initiation factor IF-2 | 1.0226±0.0196 |
|  | LOC113215274 | XM_026434883.1 | zinc finger protein 501 | 1.0243±0.0421 |
|  | LOC113215090 | XM_026434660.1 | merozoite surface antigen 2 | 1.0247±0.0243 |
|  | LOC113216217 | XM_026435956.1 | doublesex- and mab-3-related transcription factor A2 | 1.0062±0.0107 |
|  | LOC113205786 | XM_026421537.1 | zinc finger BED domain-containing protein 4 | 1.0093±0.0160 |
|  | LOC113211779 | XM_026430255.1 | histone H3v1 | 1.0094±0.0163 |
|  | LOC113215486 | XM_026435114.1 | mediator of RNA polymerase II subunit 15 | 1.0101±0.0175 |
|  | LOC113217112 | XM_026436953.1 | RING finger protein 121 | 1.2995±0.5188 |
|  | LOC113216493 | XM_026436239.1 | histone H1 | 1.0144±0.0250 |
|  | LOC113214183 | XM_026433473.1 | histidine-rich protein DDB_G0274557 | 1.0164±0.0283 |
|  | LOC113216987 | XM_026436822.1 | JNK1/MAPK8-associated membrane protein | 1.0272±0.0472 |
|  | LOC113217076 | XM_026436917.1 | KIF1-binding protein homolog | 1.0295±0.0510 |
|  | LOC113206320 | XM_026422345.1 | zinc finger BED domain-containing protein 5 | 1.0748±0.1296 |
| Cell cycle (6) | LOC113217060 | XM_026436901.1 | insulin-degrading enzyme 2 | 1.0920±0.1594 |
|  | LOC113211022 | XM_026429257.1 | E3 ubiquitin-protein ligase NHLRC1 | 1.0093±0.0162 |
|  | LOC113205179 | XM_026420690.1 | FAM10 family protein At4g22670 | 1.0206±0.0450 |
|  | LOC113212373 | XM_026431047.1 | serine proteinase stubble transcript variant X1 | 1.1129±0.1438 |
|  | LOC113205669 | XM_026421377.1 | putative inorganic phosphate cotransporter | 1.3666±0.4002 |
|  | LOC113216826 | XM_026436652.1 | ubiquitin-conjugating enzyme E2 2 | 1.0446±0.0398 |
| Others (21) | LOC113214441 | XM_026433801.1 | uncharacterized | 1.0150±0.0260 |
|  | LOC113210125 | XM_026427957.1 | immunoglobulin G-binding protein A | 1.0231±0.0288 |
|  | LOC113212197 | XM_026430809.1 | cytochrome P450 4c3 | 1.0092±0.0159 |
|  | LOC113212226 | XM_026430838.1 | uncharacterized | 1.1149±0.1991 |
|  | LOC113215541 | XM_026435180.1 | uncharacterized | 1.0869±0.1505 |
|  | LOC113218200 | XM_026438447.1 | uncharacterized | 1.1014±0.1756 |
|  | LOC113214487 | XM_026433853.1 | uncharacterized | 1.0247±0.0428 |
|  | LOC113214801 | XM_026434279.1 | uncharacterized | 1.0168±0.0291 |
|  | LOC113212001 | XM_026430563.1 | uncharacterized | 1.0318±0.0551 |
|  | LOC113215163 | XM_026434760.1 | uncharacterized | 1.0397±0.0688 |
|  | LOC113203107 | XM_026417615.1 | uncharacterized | 1.0428±0.0234 |
|  | LOC113215244 | XM_026434840.1 | uncharacterized | 1.043±0.07450 |
|  | LOC113214944 | XM_026434472.1 | uncharacterized | 1.0058±0.0100 |
|  | LOC113215191 | XM_026434805.1 | uncharacterized | 1.0059±0.0102 |
|  | LOC113217548 | XM_026437500.1 | uncharacterized | 1.0024±0.0042 |
|  | LOC113212504 | XM_026431228.1 | uncharacterized | 1.0027±0.0048 |
|  | LOC113215825 | XM_026435490.1 | uncharacterized protein K02A2.6 | 1.0029±0.0050 |
|  | LOC113212318 | XM_026430966.1 | uncharacterized | 1.0036±0.0062 |
|  | LOC113204538 | XM_026419744.1 | uncharacterized | 1.0041±0.0071 |
|  | LOC113215279 | XM_026434888.1 | uncharacterized | 1.0074±0.0128 |
|  | LOC113214739 | XM_026434202.1 | uncharacterized | 1.0114±0.0197 |
|  | LOC113211008 | XM_026429242.1 | uncharacterized | 1.0262±0.0258 |
|  | LOC113214697 | XM_026434150.1 | uncharacterized | 1.0129±0.0223 |

^1^Figures in parentheses indicate the number of genes in each category.
