# Supplementary material for: Transcriptome analysis of female western flower thrips, Frankliniella occidentalis, exhibiting neo-panoistic ovarian development
Source: PLoS One. 2022 Aug 1;17(8):e0272399. doi: 10.1371/journal.pone.0272399 (PMC9342723; doi:10.1371/journal.pone.0272399)
Supplement: S3 Table — (DOCX) [file pone.0272399.s003.docx]

**Table S3.** Annotation of 68 genes expressed only at 60 h after adult emergence (AAE) compared to expression levels at the early (0 h AAE) developmental stage in female *F. occidentalis* adults

| Category  (47) ^1^ | NCBI  Gene ID | GenBank  accession | | Annotation | RPKM |
| --- | --- | --- | --- | --- | --- |
| Structure (8) | LOC113216413 | XM_026436158.1 | ETS domain-containing protein | | 1.0067±0.0115 |
|  | LOC113215538 | XM_026435176.1 | protein starmaker | | 1.0016±0.0027 |
|  | LOC113210317 | XM_026428265.1 | integrator complex subunit 12 | | 1.6941±1.0142 |
|  | LOC113216523 | XM_026436272.1 | endocuticle structural glycoprotein SgAbd | | 1.0099±0.0171 |
|  | LOC113217831 | XM_026437898.1 | basic salivary proline-rich protein | | 1.0216±0.0246 |
|  | LOC113203658 | XM_026418447.1 | phosphopantothenoylcysteine decarboxylase subunit VHS3 | | 1.0147±0.0255 |
|  | LOC113205937 | XM_026421751.1 | serine/arginine repetitive matrix protein 1 | | 1.0081±0.0140 |
|  | LOC113213964 | XM_026433191.1 | serine/arginine repetitive matrix protein 1 | | 1.0052±0.0091 |
| Protein processing (11) | LOC113215722 | XM_026435388.1 | gamma-gliadin | | 1.0101±0.0175 |
|  | LOC113201813 | XM_026415742.1 | cell death-inducing p53-target protein 1 | | 1.0536±0.0534 |
|  | LOC113211526 | XM_026429922.1 | proline-rich protein HaeIII subfamily 1 | | 1.0526±0.0911 |
|  | LOC113211589 | XM_026429998.1 | octapeptide-repeat protein T2 | | 1.0109±0.0188 |
|  | LOC113213325 | XM_026432345.1 | ubiquitin carboxyl-terminal hydrolase 36 | | 1.0137±0.0238 |
|  | LOC113213336 | XM_026432359.1 | U1 small nuclear ribonucleoprotein 70 kDa | | 1.0360±0.0624 |
|  | LOC113214194 | XM_026433487.1 | vasodilator-stimulated phosphoprotein | | 1.0602±0.1043 |
|  | LOC113210122 | XM_026427952.1 | protein antagonist og-like heterochomatin protein | | 1.0425±0.0736 |
|  | LOC113210219 | XM_026428096.1 | protein atonal | | 1.0235±0.0408 |
|  | LOC113215678 | XM_026435345.1 | thymidylate kinase | | 1.0098±0.0170 |
|  | LOC113215719 | XM_026435386.1 | ubiquitin-conjugating enzyme E2-16 kDa | | 1.0143±0.0248 |
| Gene regulation (11) | LOC113210852 | XM_026429021.1 | allergen Cr-PI | | 1.0027±0.0046 |
|  | LOC113211028 | XM_026429265.1 | KRAB-A domain-containing protein 2 | | 1.0481±0.0833 |
|  | LOC113209254 | XM_026426677.1 | DNA endonuclease RBBP8-like | | 1.0673±0.1165 |
|  | LOC113217197 | XM_026437023.1 | phosphoribosylformylglycinamidine cyclo-ligase | | 1.0880±0.1525 |
|  | LOC113217232 | XM_026437052.1 | inositol hexakisphosphate and diphosphoinositol-pentakisphosphate kinase 2 | | 1.0672±0.1164 |
|  | LOC113215917 | XM_026435577.1 | zinc finger protein 26 | | 1.0268±0.0269 |
|  | LOC113202596 | XM_026416897.1 | gustatory and odorant receptor 63a | | 1.0041±0.0071 |
|  | LOC113216161 | XM_026435909.1 | translation initiation factor IF | | 1.0110±0.0191 |
|  | LOC113216830 | XM_026436660.1 | general transcription factor II-I repeat domain protein | | 1.0263±0.0456 |
|  | LOC113218190 | XM_026438436.1 | anti-sigma-I factor RsgI2 | | 1.0055±0.0096 |
|  | LOC113202631 | XM_026416962.1 | nuclease HARBI1 | | 1.0078±0.0135 |
| Others (37) | LOC113210531 | XM_026428578.1 | uncharacterized | | 1.0037±0.0064 |
|  | LOC113214996 | XM_026434537.1 | protein FAM133A | | 1.0070±0.0121 |
|  | LOC113201962 | XM_026415978.1 | uncharacterized | | 1.3469±0.2718 |
|  | LOC113218381 | XM_026438709.1 | uncharacterized | | 1.0029±0.0050 |
|  | LOC113203833 | XM_026418732.1 | uncharacterized | | 1.0094±0.0094 |
|  | LOC113204701 | XM_026419966.1 | uncharacterized | | 1.0320±0.0553 |
|  | LOC113205840 | XM_026421608.1 | uncharacterized | | 1.0010±0.0017 |
|  | LOC113211785 | XM_026430260.1 | uncharacterized | | 1.0075±0.0131 |
|  | LOC113212002 | XM_026430564.1 | uncharacterized | | 1.0127±0.0220 |
|  | LOC113212270 | XM_026430895.1 | uncharacterized | | 1.0174±0.0302 |
|  | LOC113212325 | XM_026430972.1 | uncharacterized | | 1.0012±0.0021 |
|  | LOC113212874 | XM_026431720.1 | uncharacterized | | 1.0254±0.0440 |
|  | LOC113213073 | XM_026432014.1 | uncharacterized | | 1.0256±0.0257 |
|  | LOC113216187 | XM_026435933.1 | uncharacterized protein K02A2.6 | | 1.0338±0.0585 |
|  | LOC113216251 | XM_026435989.1 | uncharacterized | | 1.0146±0.0252 |
|  | LOC113214466 | XM_026433835.1 | uncharacterized | | 1.0080±0.0080 |
|  | LOC113214602 | XM_026434038.1 | uncharacterized | | 1.0043±0.0075 |
|  | LOC113214631 | XM_026434073.1 | uncharacterized | | 1.0020±0.0035 |
|  | LOC113214912 | XM_026434429.1 | uncharacterized | | 1.0020±0.0035 |
|  | LOC113215033 | XM_026434590.1 | uncharacterized | | 1.0496±0.0495 |
|  | LOC113215258 | XM_026434861.1 | uncharacterized | | 1.0055±0.0095 |
|  | LOC113215455 | XM_026435088.1 | uncharacterized | | 1.0141±0.0244 |
|  | LOC113215456 | XM_026435089.1 | uncharacterized | | 1.0209±0.0362 |
|  | LOC113202760 | XM_026417145.1 | uncharacterized | | 1.0059±0.0103 |
|  | LOC113203543 | XM_026418297.1 | uncharacterized | | 1.0125±0.0217 |
|  | LOC113215596 | XM_026435236.1 | uncharacterized | | 1.0505±0.0666 |
|  | LOC113206868 | XM_026423142.1 | uncharacterized | | 1.0127±0.0220 |
|  | LOC113206964 | XM_026423276.1 | uncharacterized | | 1.0036±0.0063 |
|  | LOC113207325 | XM_026423841.1 | uncharacterized | | 1.0389±0.0243 |
|  | LOC113213654 | XM_026432774.1 | uncharacterized protein ENSP00000383309 | | 1.0104±0.0180 |
|  | LOC113209904 | XM_026427668.1 | COMPASS component SPP1-like | | 1.0188±0.0326 |
|  | LOC113213686 | XM_026432817.1 | uncharacterized | | 1.0084±0.0146 |
|  | LOC113214024 | XM_026433268.1 | uncharacterized | | 1.0053±0.0091 |
|  | LOC113214132 | XM_026433407.1 | uncharacterized | | 1.0555±0.0481 |
|  | LOC113211236 | XM_026429553.1 | uncharacterized | | 1.0421±0.0729 |
|  | LOC113211336 | XM_026429687.1 | uncharacterized | | 1.0167±0.0072 |
|  | LOC113210242 | XM_026428122.1 | uncharacterized | | 1.0186±0.0322 |

^1^Figures in parentheses indicate the number of genes in each category.
