# Supplementary material for: Transcriptome analysis of female western flower thrips, Frankliniella occidentalis, exhibiting neo-panoistic ovarian development
Source: PLoS One. 2022 Aug 1;17(8):e0272399. doi: 10.1371/journal.pone.0272399 (PMC9342723; doi:10.1371/journal.pone.0272399)
Supplement: S4 Table — (DOCX) [file pone.0272399.s004.docx]

**Table S4.** Highly ( > 8-fold) suppressed genes at mid (36 h after adult emergence) and late (60 h after adult emergence) ovarian development stages compared to expression levels in the early (0 h after adult emergence) developmental stage in female *F. occidentalis* adults

| Category (35) | NCBI  Gene ID | GenBank  accession | Annotation | 36 h | | 60 h | |
| --- | --- | --- | --- | --- | --- | --- | --- |
|  |  |  |  | RPKM | Log_2_ Fc | RPKM | Log_2_ Fc |
| Structure (16) | LOC113208180 | XM_026425071.1 | glycine-rich cell wall structural protein | 0.15 | -3.44 | 0.04 | -3.77 |
|  | LOC113205908 | XM_026421701.1 | sarcalumenin | 0.06 | -3.22 | 0.00 | -3.43 |
|  | LOC113215239 | XM_026434835.1 | flightin | 0.96 | -3.06 | 0.64 | -4.13 |
|  | LOC113207997 | XM_026424844.1 | cuticle protein 70 | 0.47 | -3.23 | 0.06 | -4.60 |
|  | LOC113207688 | XM_026424343.1 | cuticle protein 63 | 0.36 | -3.19 | 0.05 | -4.22 |
|  | LOC113203515 | XM_026418251.1 | cuticle protein 1 | 0.16 | -2.83 | 0.00 | -3.36 |
|  | LOC113203516 | XM_026418252.1 | cuticle protein 1 | 0.52 | -4.14 | 0.01 | -5.82 |
|  | LOC113207694 | XM_026424349.1 | larval cuticle protein F1 | 0.25 | -4.10 | 0.00 | -4.92 |
|  | LOC113211940 | XM_026430488.1 | larval cuticle protein F1 | 0.25 | -4.06 | 0.00 | -4.88 |
|  | LOC113202389 | XM_026416582.1 | larval cuticle protein A3A | 0.13 | -2.97 | 0.01 | -3.36 |
|  | LOC113202386 | XM_026416580.1 | larval cuticle protein A2B | 0.78 | -4.07 | 0.29 | -5.70 |
|  | LOC113209338 | XM_026426787.1 | collagen alpha-2(IV) chain | 0.22 | -3.09 | 0.02 | -3.76 |
|  | LOC113206937 | XM_026423231.1 | collagen alpha-1(III) chain | 0.33 | -2.67 | 0.16 | -3.27 |
|  | LOC113204878 | XM_026420251.1 | endocuticle structural glycoprotein | 0.38 | -3.64 | 0.04 | -4.76 |
|  | LOC113201709 | XM_026415579.1 | myosin heavy-chain muscle | 1.76 | -2.04 | 1.43 | -3.12 |
|  | LOC113205220 | XM_026420745.1 | adult-specific cuticular protein ACP | 0.09 | -4.30 | 0.03 | -4.48 |
| Gene regulation (8) | LOC113216684 | XM_026436480.1 | neurochondrin | 0.00 | -4.07 | 0.00 | -4.07 |
|  | LOC113216440 | XM_026436191.1 | GTP cyclohydrolase 1 | 1.68 | -2.49 | 1.47 | -3.19 |
|  | LOC113201793 | XM_026415711.1 | glutathione S-transferase 1 | 0.58 | -2.64 | 0.44 | -3.09 |
|  | LOC113218194 | XM_026438440.1 | zinc finger protein 512B | 1.11 | -4.28 | 0.11 | -7.60 |
|  | LOC113203482 | XM_026418198.1 | deoxynucleoside triphosphate triphosphohydrolase SAMHD1 | 0.18 | -2.79 | 0.02 | -3.32 |
|  | LOC113209658 | XM_026427304.1 | F-box/LRR-repeat protein 14 | 0.73 | -3.03 | 0.55 | -3.63 |
|  | LOC113201987 | XM_026416013.1 | forkhead box protein K1 | 0.07 | -4.14 | 0.05 | -4.19 |
|  | LOC113217596 | XM_026437553.1 | BRD4-interacting chromatin-remodeling complex-associated protein | 0.58 | -4.10 | 0.05 | -5.87 |
| Others (11) | LOC113209752 | XM_026427428.1 | uncharacterized | 0.73 | -2.38 | 0.50 | -3.17 |
|  | LOC113209220 | XM_026426635.1 | uncharacterized | 0.01 | -4.30 | 0.02 | -4.27 |
|  | LOC113207924 | XM_026424691.1 | uncharacterized | 0.27 | -3.72 | 0.04 | -4.47 |
|  | LOC113205973 | XM_026421819.1 | uncharacterized | 0.40 | -2.37 | 0.19 | -3.08 |
|  | LOC113205261 | XM_026420807.1 | uncharacterized | 0.79 | -2.96 | 0.51 | -3.88 |
|  | LOC113203955 | XM_026418920.1 | uncharacterized | 0.56 | -2.47 | 0.22 | -3.62 |
|  | LOC113202387 | XM_026416581.1 | uncharacterized | 0.30 | -3.29 | 0.01 | -4.23 |
|  | LOC113207698 | XM_026424353.1 | calphotin | 0.81 | -4.34 | 0.19 | -6.36 |
|  | LOC113214392 | XM_026433740.1 | allergen Cr-PIe | 0.11 | -3.20 | 0.08 | -3.31 |
|  | LOC113214583 | XM_026434011.1 | immunoglobulin domain and leucine-rich repeat-containing protein | 0.41 | -2.84 | 0.18 | -3.60 |
|  | LOC113207272 | XM_026423753.1 | fibrous sheath CABYR-binding protein | 0.00 | -3.70 | 0.00 | -3.70 |
