# Supplementary material for: Transcriptome analysis of female western flower thrips, Frankliniella occidentalis, exhibiting neo-panoistic ovarian development
Source: PLoS One. 2022 Aug 1;17(8):e0272399. doi: 10.1371/journal.pone.0272399 (PMC9342723; doi:10.1371/journal.pone.0272399)
Supplement: Attachment — Submitted filename: Reviewers comment-PONE-D-22-15697.docx [file pone.0272399.s005.docx]

Reviewer’s comment

Title: Transcriptome analysis of female western flower thrips, *Frankliniella occidentalis*, exhibiting neo-panoistic ovarian development.

In this study, the authors Choi and Kim have done transcriptome analysis of pest western flower thrips *Frankliniella* *occidentalis* at mid (36 h after adult emergence (AAE)) and late (60 h AAE) ovarian developmental stages using RNA sequencing (RNASeq) technology. More than 120 million reads per replication were matched to » 15,000 *F. occidentalis* genes. Almost 500 genes were expressed at each mid and late ovarian developmental stage. Differentially expressed genes (DEGs) were associated with metabolic pathways and protein and nucleic acid biosynthesis. In both ovarian developmental stages, vitellogenin, mucin, and chorion genes were highly (> 8-fold) expressed. Endocrine signals associated with ovarian development were further investigated from the DEGs. Insulin and juvenile hormone signals were upregulated only at 36 h AAE, whereas the ecdysteroid signal was highly maintained at 60 h AAE.

The thrip is the primary vector for a few plant viruses such as tomato spotted wilt virus (TSWV) and impatiens necrotic spot virus (INSV). The study is interesting and vital as the thrips can destroy many crops worldwide by feeding on them and by spreading the plant viruses. There are few reports on the transcriptomic analysis of the thrips during virus infection, but that of on reproduction of *F. occidentalis* is rare in the literature. However, the discussion part is important here. The RNA/gene expression results are straight forward but how these differentially expressed genes contribute to a faster reproduction rate needs to be discussed in detail. Here are few suggestions which might improve the value of the manuscript.

1. Discussion: How these results help in the control of the pest directly or indirectly needs to be explained. What is the significance of expressed genes? The authors may consider including literature support on this aspect.
2. Resolution of figures 3, 4 and 5 to be improved.
3. Line 98-99: Mention that the total RNA extracted was from the whole thrips.
4. As this article is on the reproduction of thrips and hence, more detail related to reproduction can be included in the introduction. How fast the pest can develop into an adult (Moritz et al. 2004. Virus Res., 100 pp. 143-149), etc.
5. The transcriptomics results need not necessarily correlate to protein expression (PMID: 26085669). Authors may consider including it in the discussion.
