# Supplementary material for: Transcriptome analysis of female western flower thrips, Frankliniella occidentalis, exhibiting neo-panoistic ovarian development
Source: PLoS One. 2022 Aug 1;17(8):e0272399. doi: 10.1371/journal.pone.0272399 (PMC9342723; doi:10.1371/journal.pone.0272399)
Supplement: Attachment — Submitted filename: Choi et al., 2022_.docx [file pone.0272399.s006.docx]

The article by Choi et al. summarizes ovarian development and associated transcriptomes of *F. occidentalis* at various stages of oocyte development (0 h AAE, 36 h AAE, and late 60 h AAE) and compares the differentially expressed genes. The study is technically well performed and described in the manuscript, yet there are some concerns that should be addressed before it can be reconsidered for publication.

1. Why do authors choose to perform RNA extraction from whole female adult *F. occidentalis* instead of just from the ovarioles?

2. In Figure 1, the authors have only shown one stage (3-day-old female adults) of ovariole structure. Since the authors are focusing on all three stages ( 0h, 36 h, and 60 h AAE) and are also measuring the ovariole length, it is important to show all stages of ovariole development.

3. For Figure 2B, it might be better to include volcano plots with differentially expressed genes pattern (36 vs 0, 60 vs 0, 36 vs 60) and top candidate gene names to guide readers to follow the changes in gene expression.

4. In Figure 3, it is difficult to follow the entire stretch of the graph. Importantly, the labels are not clearly legible. Authors can show only the pathways that are up or down-regulated between 60 vs 36 instead of showing the number of genes.

5. Figures 4 & 5: Authors may consider merging these two figures into one figure. Also, is there a particular reason for including RPKM values on the main figure?
